# Notices

**Published:** 1868-08

**Authors:** 


EPILEPSY. In Cornell's "Guardian of Health," for June, (No. 4 Hayward Pl. Boston), is a valuable paper on this distressing malady. Price 25 cts.

ALASKA. The Hon. E. D. Morgan, Senator from this city at Washington, has sent us an 8vo, document of 361 pp. relative to Russian America, containing Mr. Sumner's admirable and exhaustive speech on the importance and necessity of the acquisition, giving a full description of the climate, soil, productions, &c. &c.

NECRNOSOZOIC, or "The Dead Made Alive," being a new Process for the Preservation of the Human Body; by Clark & Co. 713 Broadway, New York, by simply washing it, it is preserved from visible change in features, form and color, for many months, and for aught we know for an indefinite period; the body is not mutilated in any way, no injections are made in the veins; no cut is made on the person, while it is left thoroughly disinfected. These simple facts are attested by the most eminent medical men in America; among these are Dr. Delafield, Prof. Jas. R. Wood, Dr. Doremus, Flint, and others. Physicians, editors, and many men of intelligence and culture, have looked upon this most perfect embalming process, discovered by Mr. W. R. C. Clark, with wonder and admiration. Circulars will be sent free of charge, by addressing as above.

CHROMOGRAPHY, or "Color Drawing," by which the fac simile of a painting, which could not be bought for a thousand dollars, can be reproduced for ten; and as much alike as one lithograph is like another, containing all the various colors and shadings of the original; this is one of the most important achievements in the art-world of the present century, and for which all the lovers of the beautiful are indebted to the indomitable enterprize and wonderful steadiness of purpose of Louis Prang of Boston, Mass. Next to the Christian Religion, and the purifying, elevating influence of woman, music and sculpture and painting are the great civilizers and elevators of the race; and it need not be for long, when the handy work of Louis Prang shall ornament the walls of every dwelling, when the humanizing influences of his Chromographs shall pervade every household, and the culture of the beautiful shall be inaugurated in every family in the land.

IMPORTANT TO HOUSE OWNERS, who want to live comfortably, healthfully and long. How many of our readers gave a second thought to the July article on "Warming Dwellings," and yet it is of more importance than who shall be the next President of the United States, because it is of vital practical interest to every man who has a house to live in, as it involves the question "How shall I best warm and ventilate my house at the least cost, next winter?" Heating houses with warm air or steam, is now coming in fashion. Ruttan's system of house warming requires one-half less coal, besides giving a more perfect ventilation, and keeping the floor the warmest part of the room. In a cold winter's day in a New York dwelling, where the ceilings are twelve feet high, there is many times a difference of thirty degrees in the temperature of the air, at the floor and the ceiling; the air at the ceiling, which we do not breathe, is the warmest and purest in the room, while the air at the floor, which we do breathe, is the coldest and the most impure. Ruttan's system of House Warming reverses this, and uses one-half less coal than is required for steam or hot air heating. During the first week in August J. W. Jewell, Professor of the natural sciences in the Illinois State Normal University, will be in New York city to give information on the subject to builders, architects and gentlemen; or address W. A. Pennell & Co., Normal, Illinois.

BALDWIN, the Clothier, will pay special attention to Boys' Clothing the coming fall and winter; largest assortment—one price—lowest in the city. C. O. D.

